# A Case Report on the Progression of Myeloid Sarcoma to Form Multiple Metastatic Deposits without Developing Acute Myeloid Leukaemia

**DOI:** 10.1155/2015/162154

**Published:** 2015-09-30

**Authors:** Sunita Kohli, Mark Lee, Scott Marshall

**Affiliations:** ^1^City Hospitals Sunderland and St. Benedict's Hospice, St. Benedict's WayRyhope SR2 0NY, UK; ^2^City Hospitals Sunderland, Sunderland Royal Hospital, Kayll Road, Sunderland SR4 7TP, UK

## Abstract

**Introduction:**

Myeloid sarcomas (MS) are rare tumours occurring at extramedullary sites. They are usually associated with other haematology disorders such as acute myeloid leukaemia, myelodysplastic syndrome, and chronic myeloproliferative neoplasms. They frequently occur with a diagnosis of acute myeloid leukaemia (AML) or with relapse of preexisting disease. Patients with myeloid sarcomas without history or evidence of myeloid leukaemia typically progress to form AML.

**Case Presentation:**

A case report of a patient diagnosed with an isolated myeloid sarcoma that rarely did not transform to AML but instead spread to form multiple myeloid sarcomas throughout the body.

**Discussion:**

This case identifies the risk of metastatic spread of these tumours rather than the development of AML which is poorly documented in the literature, due to the rarity of cases, and may be significant in the investigation and management of isolated myeloid sarcomas. This case highlights the need for clinicians to consider repeat cross-sectional imaging to investigate unexplained clinical decline or symptoms, when there is no sign of AML progression and to consider radiotherapy treatment early.

## 1. Introduction

Myeloid sarcoma (MS) is a rare tumour containing immature myeloid cells or blasts occurring in an extramedullary site, associated with acute myeloid leukaemia (AML), myelodysplastic syndrome (MDS), or chronic myeloproliferative neoplasms. Myeloid sarcoma usually presents with AML, following diagnosis, or as relapse of previous disease and is reported in 2–9% of AML cases. The term isolated myeloid sarcoma is considered where tumour is found without any current evidence of blood or bone marrow involvement or previous history of myeloid disease. Most cases of isolated myeloid sarcomas will progress to AML within the year. Myeloid sarcomas can be found in various sites, most commonly in soft tissue, bone, and lymph nodes [1–4].

This case reports a patient with isolated MS which fails to progress with blood or bone marrow involvement but disseminates to form multiple myeloid sarcomas throughout the body within two years of diagnosis. We present this case to highlight the risk of metastatic spread of these tumours rather than development of acute leukaemia which is poorly documented in the medical literature and may be significant in the investigation and management of the patient.

## 2. Case Presentation

**Figure 1:**
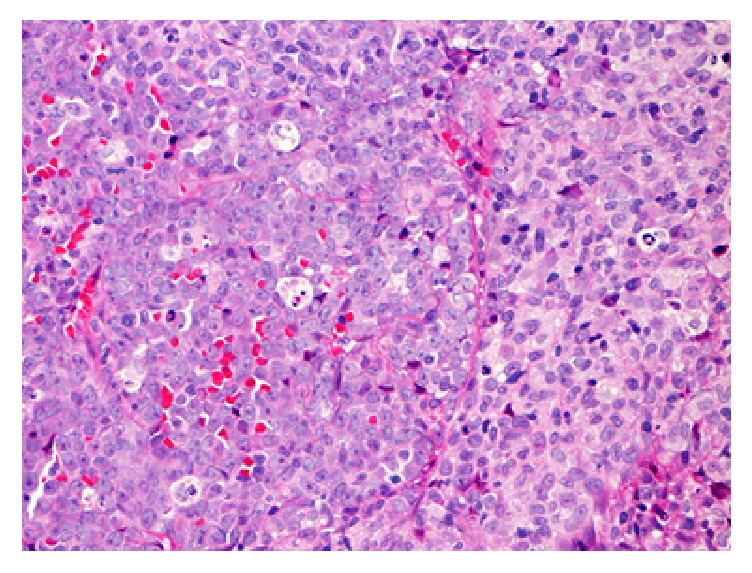
A morphology slide showing many neoplastic cells.

A 53-year-old male, with worsening chronic back pain but no focal neurology, was referred for a haematology opinion following a magnetic resonance imaging (MRI) scan which showed a large extradural soft tissue mass from T5 to T9 that was displacing the spinal cord, pressing on his descending aorta, and extending through his left intercostal muscles. Biopsy with morphology and immunohistochemical staining of this paraspinal mass confirmed a myeloid sarcoma. Many of the neoplastic cells found on morphology were myeloblasts with eosinophilic cytoplasm, fine chromatin, and prominent nucleoli (left side of Figure 1). These expressed mostly CD33 and CD34 immunohistochemistry with some positive for CD117 and myeloperoxidase. There were also clusters of myeloid cells showing monocytic differentiation with ovoid nuclei and abundant pale cytoplasm (right side of Figure 1). These expressed CD33, CD4, and CD14 but were negative for CD34.  Box 1 shows full results of the immunohistochemical stains performed with notably positive stains for CD68, CD43, CD34, and scattered TdT and Ki67 positivity [1, 3–5].

The initial bone marrow biopsy contained approximately 5% nucleated blasts; thus the patient was diagnosed with isolated myeloid sarcoma. This was treated as acute myeloid leukaemia and the patient entered the AML 17 clinical trial following discussion with the trial chief investigator, receiving Daunorubicin and Cytarabine chemotherapy. The AML 17 trial required monitoring with bone marrow response. As the patient had an essentially normal bone marrow, monitoring could not be possible and he was therefore withdrawn after the first course of the treatment.

**Figure 2:**
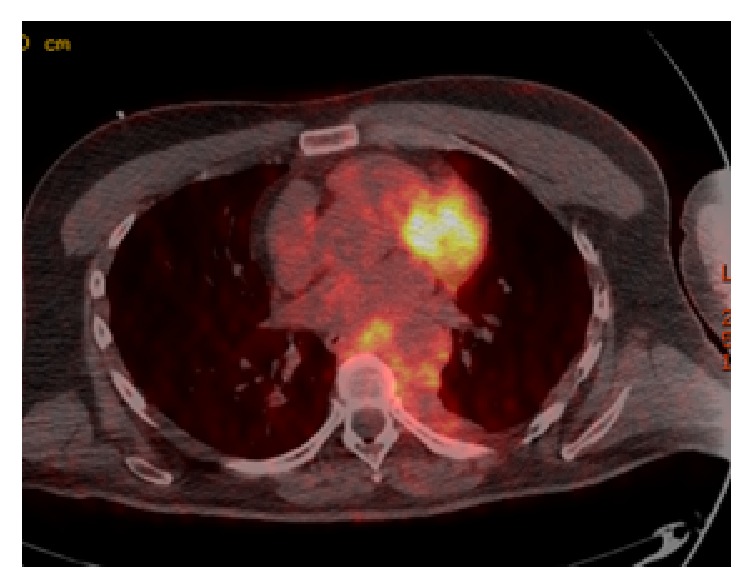
Baseline PET Scan following one course of chemotherapy showing metabolic activity in the mass. Note after clarification from nuclear medicine radiologist: myocardial uptake is present as patient did not comply with 6-hour glucose fasting prior to scan.

A repeat MRI following the first course of chemotherapy showed minimal response, with less than 50% reduction in size of the mass. A baseline positron emission tomography (PET) scan performed at a similar time demonstrated metabolic activity of this paravertebral mass (shown in Figure 2). Due to the poor initial response and its proximity to the central nervous system (CNS) empirical intrathecal Cytarabine was added to each course of chemotherapy.

During the second intrathecal chemotherapy administration, cerebrospinal fluid (CSF) taken identified myeloid blasts. The patient was subsequently changed to FLAG-IDA chemotherapy, the gold standard therapy at the time, for high risk leukaemia.

An MRI scan following the first course of FLAG-IDA chemotherapy showed a good response with 50% reduction in size of the mass, with subsequent CSF samples showing no signs of further blasts. The intended plan of weekly intrathecal Cytarabine became difficult for the patient to tolerate and was hindered by episodes of neutropenic sepsis. However, as FLAG-IDA chemotherapy contained high dose Cytarabine with more effective CNS penetration the need for intrathecal Cytarabine was reduced. Two further CSF samples following FLAG-IDA chemotherapy showed no blasts and therefore intrathecal therapy was discontinued.

**Figure 3:**
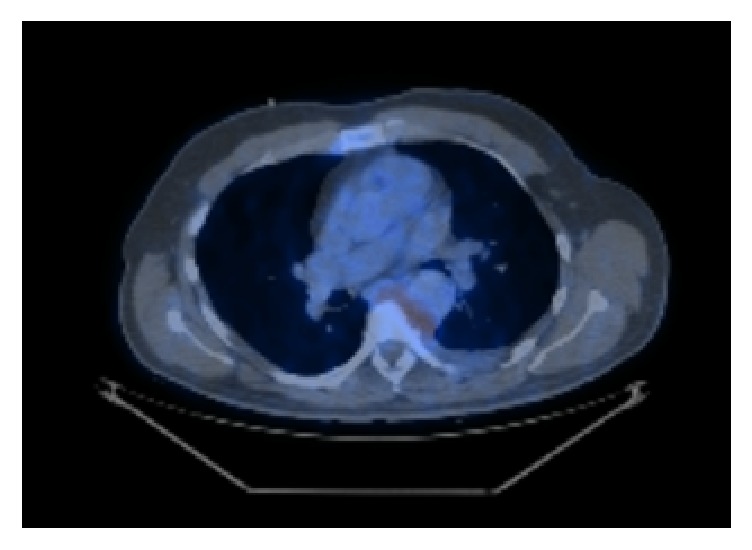
PET scan following four courses of chemotherapy still demonstrating metabolic activity.

Following three courses of FLAG-IDA chemotherapy a repeat PET scan was performed (shown in Figure 3). Although this demonstrated some decrease in volume of the mass, it showed high metabolic activity. As there was no significant change to the mass following four courses of chemotherapy intended to treat acute myeloid leukaemia, palliative radiotherapy was considered to help control local disease. Chemotherapy was to only be reconsidered if disease progression occurred.

**Figure 4:**
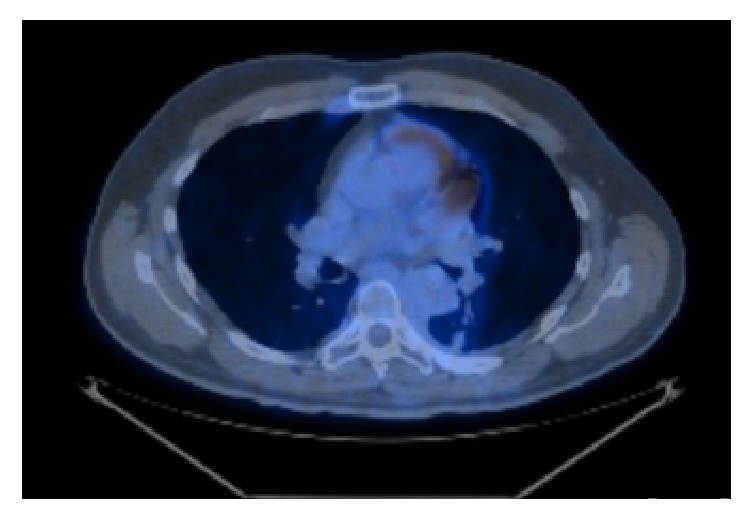
PET scan following palliative radiotherapy now metabolically negative. Note after clarification from nuclear medicine radiologist: myocardial uptake is present as patient did not comply with 6-hour glucose fasting prior to scan.

Following radiotherapy treatment aimed at the site of the mass (details shown in Table 1) a repeat PET scan showed that the myeloid sarcoma was now metabolically negative (Figure 4). The PET scan did demonstrate metabolic activity in the lung but was thought to reflect postradiotherapy change rather than metastases. Radiologist advice was also obtained for the cause of myocardial uptake, likely due to poor patient compliance of the six-hour glucose fasting required prior to scan. Blood tests at this point showed no evidence of transformation to acute leukaemia.

This patient presented a few months later to hospital with a severe headache. To exclude subarachnoid haemorrhage, a lumbar puncture was performed. CSF fluid identified myeloblasts suggestive of a relapse of myeloid sarcoma in the CNS. Blood samples at this point did not identify progression to acute leukaemia. Craniospinal radiotherapy was therefore given (details shown in Table 2).

**Figure 5:**
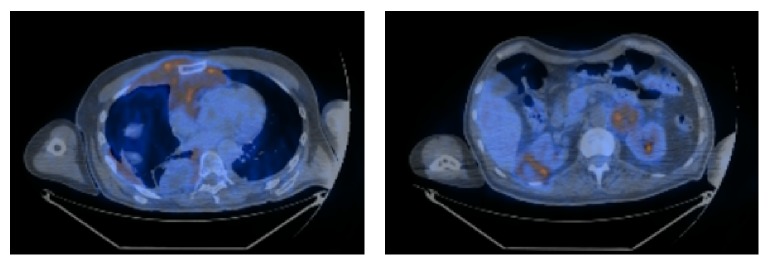
Slides from final PET scan showing metastatic spread with metabolic activity in multiple sites of the body.

Months later, the patient presented with symptoms of general decline and was referred for palliative input. Blood films still did not demonstrate any AML transformation although no bone marrow biopsy was performed. After deliberation the patient had a further PET scan, six months after the previous scan (slides shown in Figure 5). This PET scan demonstrated a large soft tissue mass infiltrating the epicardium anteriorly, right intercostal space, and the right pectoralis musculature. Further FDG positive disease was found above and below the diaphragm with supraclavicular and retroperitoneal lymphadenopathy and at the level of C4–C6 of the cervical spine. There was also a left para-aortic nodal mass encroaching the left renal hilum and a soft tissue mass encasing the distal abdominal aorta. Further FDG uptake was found in the head of the pancreas, the right lobe of the liver, the right groin, and the medial aspect of the left thigh and was bilaterally infiltrating the pleura fluid. This PET scan described widely disseminated malignancy, confirming massive disease progression. The patient was too unwell for chemotherapy and died shortly later of metastatic myeloid sarcoma.

## 3. Discussion

Here, we present a case of disseminated extramedullary myeloid sarcoma without bone marrow or blood involvement, initially involving soft tissue but also progressing to involve the CNS.

There are many case reports on diagnosing and treating isolated MS. To our knowledge, very few other case reports demonstrate this similar progression of isolated myeloid sarcoma to form multiple metastases throughout the body leading to the cause of death without any progression to leukaemia [6–9].

Although this patient had close blood film monitoring, the metastases of the myeloid sarcoma could have easily been missed without the further imaging. This highlights the importance of monitoring for progression of myeloid sarcomas, not only with blood films or bone marrow biopsies (looking for AML conversion) but also with PET scan imaging [1].

There is limited evidence on the prognosis of myeloid sarcomas, with most conclusions from retrospective studies or case comparisons. Some case reports identifying isolated myeloid sarcoma with no progression to AML were lost to follow-up during the study period with the progression of disease unknown [2, 10, 11].

Yamauchi and Yasuda [2] found isolated MS had higher rates of progression to AML with localised treatment versus systemic chemotherapy. Tsimberidou et al. [12] reported survival was nonsignificantly longer with isolated MS after chemotherapy versus acute myeloid leukaemia. Movassaghian et al. [13] analysed data and concluded overall survival in isolated MS was better than in AML; however, outcome varied depending on the site of involvement with MS. Systemic chemotherapy as per AML treatment is the current recommended management followed by hematopoietic stem cell transplantation [2, 10–13].

Although the role of radiotherapy as additional treatment for the management of myeloid sarcoma has not been fully established, the data is promising. Bakst et al. [1] analysed treatment response of chloromas to radiotherapy treatment (RT) and found that following RT local disease was very well controlled. Tsimberidou et al. [15] also agreed that the addition of radiotherapy with chemotherapy for nonleukemic granulocytic sarcoma was associated with increased failure-free survival time, particularly in those with CNS involvement; however, it did not affect overall survival. In our case radiotherapy was only considered after the mass had failed to respond to chemotherapy. If radiotherapy was given earlier could this have delayed the relapse of disease in this case [3, 14, 15]?

## 4. Conclusion

There are limited studies on prognosis of myeloid sarcoma due to the rarity of cases and lack of randomised controlled trials. Not only is the progression of disease by development of AML, but also, as highlighted in this case, further metastatic deposits can occur. This risk should prompt clinicians to consider repeat cross-sectional imaging to investigate unexplained symptoms or clinical decline when blood monitoring shows no progression to AML.

Currently evidence supports the management of isolated MS with systemic chemotherapy regimens used for treatment of acute myeloid leukaemia; however, the role of radiotherapy should be explored further and considered earlier in treatment.

## Figures and Tables

**Box 1:**
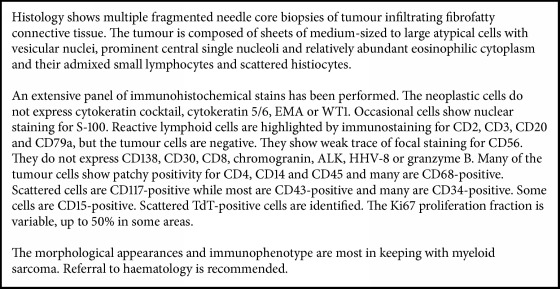
Immunohistochemical stain.

**Table 1:** First radiotherapy treatment.

Radiotherapy treatment	Fractions	Site
30 Gy	15	Initial myeloid sarcoma mass T6–T10

**Table 2:** Second radiotherapy treatment.

Radiotherapy treatment	Fractions	Site
30 Gy	15	Whole brain
